# Glenolabral Articular Disruption (GLAD) Is Not Associated with Worse Outcomes or Higher Instability Recurrence after Arthroscopic Bankart Repair—A Matched-Pair Analysis

**DOI:** 10.3390/jcm13175067

**Published:** 2024-08-27

**Authors:** Romed P. Vieider, Sebastian Siebenlist, Jose C. Sanchez, Selina Heil, Anja Wackerle, Lorenz Fritsch, Bastian Scheiderer, Maximilian Hinz, Lucca Lacheta

**Affiliations:** Department of Sports Orthopaedics, Technical University of Munich, Ismaninger Str. 22, 81675 Munich, Germany; romed.vieider@hotmail.com (R.P.V.); jose.sanchez@tum.de (J.C.S.); selina.heil@tum.de (S.H.); anja.wackerle@tum.de (A.W.); bastian.scheiderer@tum.de (B.S.); maximilian.hinz@tum.de (M.H.); lucca.lacheta@tum.de (L.L.)

**Keywords:** Bankart, arthroscopy, shoulder, shoulder instability, GLAD lesion, labral lesion, glenolabral articular disruption, arthroscopic Bankart repair

## Abstract

**Background:** This study aimed to compare clinical outcomes and recurrence of instability after arthroscopic Bankart repair (ABR) in patients with anterior shoulder instability, with and without a GLAD lesion, while distinguishing between primary and recurrent instability. **Methods:** Consecutive patients who underwent isolated ABR between January 2012 and December 2021 were included. Patients with a concomitant GLAD lesion were matched in with patients without a GLAD lesion according to the following criteria: age, sex, BMI, follow-up time, and primary versus recurrent instability. At minimum two-year follow-up, the clinical outcome (Rowe score, redislocation rate) and the functional outcome, including the American Shoulder and Elbow Surgeons (ASES) score, Western Ontario Shoulder Instability Index (WOSI), Oxford Shoulder Instability Score (OSIS), satisfaction (1–10 scale, 0 = unsatisfied, 10 = very satisfied), and Visual Analogue Scale (VAS), were compared between groups. **Results:** In total, 28 patients (14 GLAD vs. 14 Bankart; age: 32.5 ± 13.0 years; sex: 92.9% male; BMI: 24.6 ± 2.2) were included 6.9 ± 2.8 (2–11) years after isolated ABR (follow-up rate 63.6%). Clinical and functional outcome did not differ significantly between patients with versus without GLAD lesions (ASES score: 100 [96.5–100] vs. 97.5 [93.3–100], *p* = 0.27); WOSI (%): 9.0 [3.7–24.5] vs. 3.8 [0.8–8.9], *p* = 0.22; Rowe score: 90.0 [75.0–100] vs. 95.0 [78.8–100], *p* = 0.57; OSIS: 46 [44.7–48] vs. 46 [43.0–48], *p* = 0.54; satisfaction: 8.9 ± 1.4 vs. 8.0 ± 1.4, *p =* 0.78; VAS 0 [0–1.3] vs. 0 [0–1.0]. In both groups, two patients (14.3%) reported a redislocation during the observation period. **Conclusions:** At short- to mid-term follow-up, ABR showed favorable outcomes, low dislocation rates, and high patient satisfaction, regardless of the presence of a GLAD lesion or primary versus recurrent instability. However, follow-up time was heterogeneous, and the follow-up rate was marginal.

## 1. Introduction

The incidence rate of anterior shoulder dislocations is estimated at 23.9 per 100,000 person-years and mainly affects young and active men [1,2,3]. Various concomitant injuries to the glenoid rim are described in the literature. The Bankart lesion is the most common injury to the anterior labrum [4]. Besides that, variations of glenolabral injuries such as the glenolabral articular disruption (GLAD) may occur [5]. The GLAD lesion is characterized by a tear that arises in the anteroinferior labrum reaching into the glenoid articular surface, which, in some cases, extends into the subchondral bone. This injury has been described to result from a forced/forceful adduction maneuver with combined abduction and external rotation [6]. The prevalence of GLAD lesions has been reported in up to 10% of cases following primary and recurrent shoulder instability [5,7].

Bankart and GLAD lesions are usually treated as a similar pathology. For both, arthroscopic Bankart repair (ABR) using suture anchors is often recommended [8,9]. Although ABR demonstrates excellent short- to mid-term outcomes, it also exhibits a recurrence of instability in up to 31% in long-term follow-ups [8,10,11]. One risk factor may be the concomitant occurrence of a GLAD lesion and the associated decrease in glenoid concavity, which could be demonstrated in clinical and biomechanical studies [12,13]. Furthermore, several previous studies have assessed if the presence of a GLAD lesion may influence functional outcomes after ABR. Studies that evaluated redislocation rates after ABR with GLAD lesions share the common characteristic that their cohorts included patients with both primary and recurrent instability [12,14,15,16].

Therefore, the objective of this study was to evaluate the clinical outcomes and redislocation rate in patients who underwent ABR for anterior shoulder instability and assess differences between patients with versus patients without a concomitant GLAD lesion. Additionally, the present study aimed to determine whether ABR following recurrent instability would be associated with inferior outcomes compared to ABR performed after primary anterior shoulder instability.

## 2. Material and Methods

Consecutive patients between 16 and 60 years of age, who underwent isolated ABR for primary anterior glenohumeral instability between January 2012 and December 2021, were eligible for inclusion. Exclusion criteria were previous shoulder surgery, concomitant glenoid or humerus fracture, posterior GLAD lesions, superior labrum anterior to posterior (SLAP) tears, humeral avulsions of the glenohumeral ligament (HAGL), Perthes lesions, anterior glenoid bone loss >12% on preoperative magnetic resonance imaging [17], and off-track Hill-Sachs lesions [18].

The presence of an isolated Bankart lesion versus patients with a concomitant GLAD lesion was determined by the surgeon intraoperatively. To reduce bias, every patient with a concomitant GLAD lesion was matched to one patient without a GLAD lesion according to the following criteria: age (±1 years), sex (m/w), BMI (±2), follow-up time (±5 months) and whether ABR was performed for primary versus recurrent instability.

### 2.1. Surgical Technique

Arthroscopic procedures were conducted in beach chair position and under combined general and regional anesthesia. Examination under anesthesia was performed to assess instability. The operative limb was secured, and the shoulder was prepped and draped. Diagnostic arthroscopy followed, utilizing standard posterior viewing portal with a 30° arthroscope. After establishing a standard anterosuperior and anteroinferior working portal, the anterior labrum was debrided and a standard Bankart repair was performed. Therefore, one anchor was placed at the 5:30 clock face position. Additionally, 2–3 anchors were placed at the 2 o’clock, 3 o’clock, and 4:30 clock face positions, depending on the severity of the labral injury [12].

**Figure 1:**
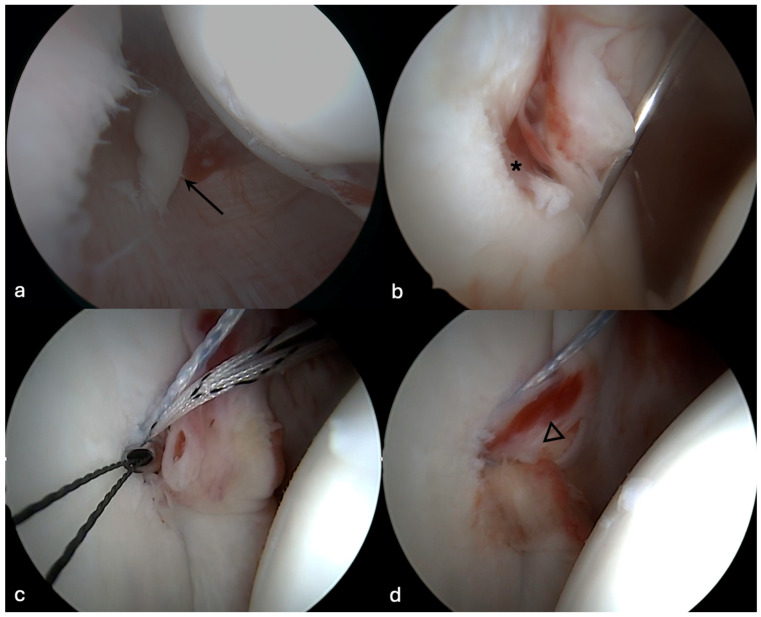
Intraoperative image of a right shoulder (**a**) through a posterior viewing portal showing the loose cartilage fragment (arrow). At the anteroinferior portion of the glenoid (**b**) a glenolabral articular disruption (GLAD lesion, *), is visible. The anchor was placed at the 5:30 o’clock position on the clock face (**c**). After fixation of the anterior labrum into the cartilage defect (**d**), the defect area is covered with labral tissue (▽).

When a GLAD lesion was present, the defect was debrided until it was shouldered. Then, the calcified layer was removed. The labrum was then repaired to the front of the glenoid to cover the cartilage defect. If the labrum was not sufficient for refixation, part of the anterior capsule was fixed into the defect (Figure 1).

### 2.2. Postoperative Rehabilitation

The rehabilitation program started with immediate postoperative sling use and limited external rotation to 30° for the initial 4 to 6 weeks. Following this, the patient gradually transitioned to active-assisted and then active range of motion exercises after weaning off the sling. Full return to activities was typically allowed within 4 to 6 months, tailored to the patient’s individual healing pace and level of activity.

### 2.3. Outcome Assessment

To compare the clinical outcomes of patients with an isolated Bankart lesion versus patients with a concomitant GLAD lesion, the American Shoulder and Elbow Surgeons (ASES) score [19], Western Ontario Shoulder Instability Index (WOSI) [20], Rowe score [21], and Oxford Shoulder Instability Score (OSIS) [22] were used at a minimum 2-year follow-up. The redislocation rate, degree of satisfaction (rated from 0 = unsatisfied to 10 = very satisfied) and Visual Analogue Scale (VAS) were compared and the rate and time to return to sports (RTS) on the preinjury level of activity was evaluated.

### 2.4. Statistical Analysis

Descriptive statistics and significance tests were conducted using SPSS 27.0 (IBM-SPSS, New York, NY, USA) and visualized through graphs and tables. Prior to analysis, data homogeneity of variance and normal distribution were assessed with the Levene test and the Shapiro–Wilk test, respectively. Continuous variables were presented as mean and standard deviation (for normally distributed data) or median and interquartile range (for non-normally distributed data). To compare pre-and postoperative data groups, comparisons were performed utilizing either a paired t-test (for normally distributed data) or Wilcoxon signed rank test (for non-normally distributed data). For comparison between the two independent groups, a student’s *t*-test or Mann–Whitney U test was performed. Due to the study’s limited case count of GLAD lesions, a priori sample size calculation was not conducted. Statistical significance was determined at *p* < 0.05.

## 3. Results

**Figure 2:**
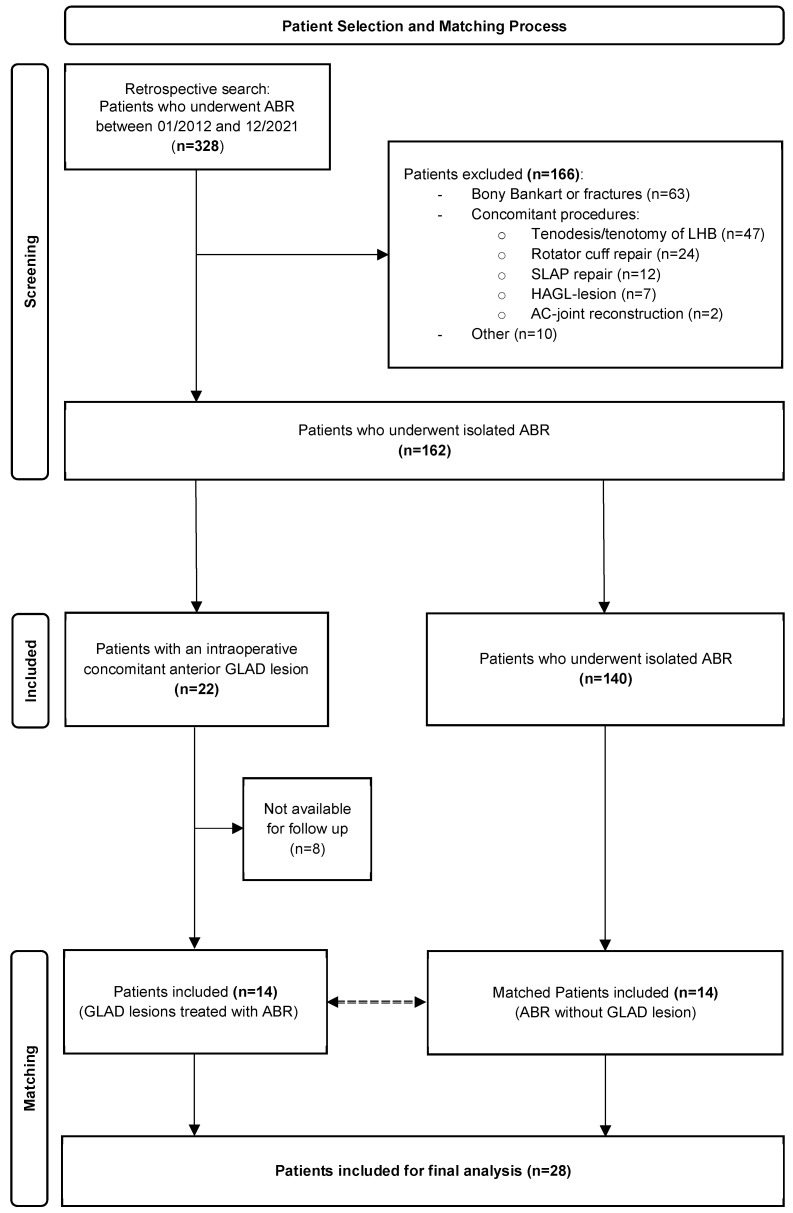
Flowchart of patient inclusion for the matched-pair analysis, comparing patients undergoing arthroscopic Bankart repair (ABR) with versus without a concomitant glenolabral articular disruption (GLAD) lesion. Acromioclivicular (AC), Humeral Avulsion of the Glenohumeral Ligament (HAGL), Long Head of the Biceps Tendon (LHB), Superior Labrum from Anterior to Posterior (SLAP).

After applying the exclusion criteria, all patients who underwent an ABR between January 2012 and December 2021 at the authors’ institution were included (*n* = 162). In total, 22 patients were diagnosed with a concomitant GLAD lesion intraoperatively and 14 patients (follow-up, 64.3%) were available for final follow-up (Figure 2). Each of these patients was matched with one patient who underwent an isolated ABR and did not have a GLAD lesion. No significant differences regarding age, BMI, follow-up time, number of placed anchors, and number of primary versus recurrent instability cases could be detected (Table 1).

### 3.1. Clinical and Functional Outcome, Return to Sport

**Figure 3:**
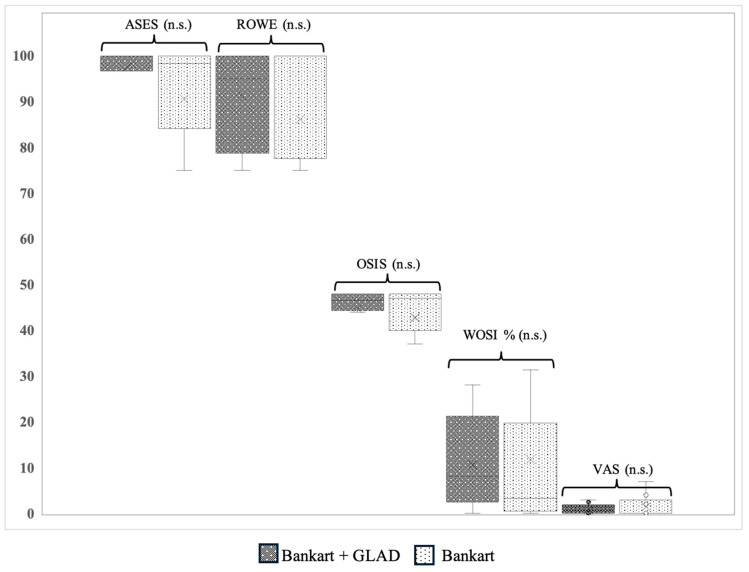
Comparison of patient-reported outcomes after ABR between patients with concomitant GLAD lesions versus without, presented in a boxplot diagram. ASES, American Shoulder and Elbow Surgeons score; GLAD, glenolabral articular disruption; ROWE, Rowe Score; n.s., not significant; OSIS, Oxford Shoulder Instability Score; VAS, Visual Analogue Scale; WOSI, Western Ontario Shoulder Instability Index.

Clinical outcomes for the study cohort were displayed as follows: median ASES of 99.2 (IQR 95.0–100), WOSI of 7.4% (IQR 1.5–11.3%), median Rowe Score of 90 (IQR 75.0–100), and median OSIS of 46 (IQR 43.3–48.0). Satisfaction levels showed a median of 9 (IQR 8–10) across both groups (Table 2). The clinical findings revealed no significant variations in any of the scores between both groups/patients with GLAD lesion versus patients without GLAD lesions (Figure 3).

In each cohort, two patients (14.3%) experienced redislocation incidents during the follow-up period (Table 3). In total, two patients (7.3%) from the Bankart group underwent subsequent revision surgery and received an open coracoid transfer according to Latarjet.

**Figure 4:**
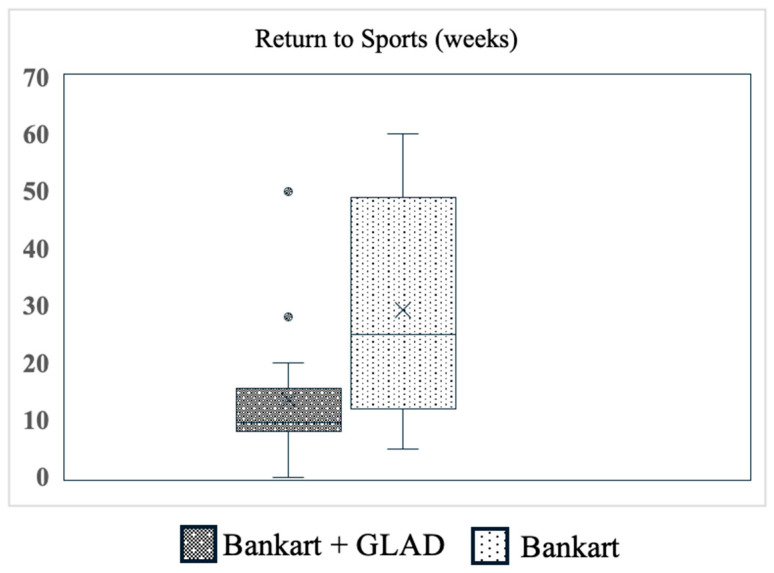
Comparison of time to return to sports (weeks) after ABR between patients with concomitant GLAD lesions versus without, presented in a boxplot diagram. The outliers are represented by the dots (°).

All patients reported that they have been able to return to sport. Considering the time to RTS, patients in the GLAD group returned to their preoperative sports significantly faster than those in the Bankart group (*p* = 0.046, Table 2, Figure 4).

### 3.2. Subgroup Analysis

When solely comparing Bankart with a concomitant GLAD versus isolated Bankart groups in cases of primary instability, no significant differences in clinical outcomes or satisfaction rates were observed between groups (Table 4). Similarly, the rates of redislocation were comparable between the two groups. Time to return to sports was significantly longer in the Bankart versus the GLAD group (24.0 (10.5–56) weeks versus 10 (9–16) weeks, *p* < 0.05).

## 4. Discussion

The most important finding of the present study was that patients with isolated Bankart lesion exhibited similar clinical and functional outcomes to those with a concomitant GLAD lesion when treated with ABR. Rates of redislocation were low, satisfaction levels were high, and the level of sports was equally favorable in both groups at a mean follow-up of almost 7 years. Furthermore, when comparing groups with primary instability prior to ABR, no differences were found in functional outcome parameters. Thus, ABR provides good clinical and functional results in patients irrespective of the presence of a GLAD lesion.

The theorem that glenohumeral instability may be caused by a decrease in the glenoid concavity due to a GLAD lesion could be proven in biomechanical studies [13,23]. A case series by Pogorzelski et al. observed a higher failure rate in patients with a concomitant GLAD lesion after ABR than in patients without [12]. Similar to the present study, other clinical studies could, however, not show that the presence of a GLAD lesion would increase the risk for instability recurrence or inferior functional outcomes after ABR [14,15,16]. A recent study by Elrick et al. compared patients treated with ABR with GLAD lesions versus without GLAD lesions and demonstrated no difference in PROMs and favorable patient satisfaction at a mean follow-up of 4.5 years [15]. However, the authors did not match their cohorts based on the number of dislocations prior to surgery. In contrast, the current study included this variable in a subgroup analysis, as comparison of primary versus recurrent anterior shoulder instability cases could bias the results. Nevertheless, no differences in the investigated outcome parameters were found between these groups after matching for primary versus recurrent instability at a 6.9 year follow-up. Additionally, the study by Elrick et al. included patients in the GLAD group who received concomitant procedures to treat the cartilage defect [15]. The current study excluded any concomitant procedures regarding the glenohumeral joint surface in the selection process.

Functional outcomes of ABR in patients with Bankart and concomitant GLAD lesions versus without, primarily focusing on return to sports, return to play, and return to competition have recently been investigated by Davey et al. Overall, over 90% of the total cohort from the referenced study returned to sports after nearly 6 months without any difference between the isolated Bankart and concomitant GLAD groups. Furthermore, 77.3% suffered an anteroinferior glenohumeral dislocation in a collision situation during a collision sport [14]. In the current study, 100% of the primary instability cases resulted from a traumatic event. In contrast, four out of 10 (40%) patients (two isolated Bankart, two Bankart and concomitant GLAD) of the recurrent instability group reported a traumatic event prior to surgery. Considering the time to return to sports, patients in the GLAD group required a shorter time (10.5 weeks) compared to the Bankart group (25.0 weeks). However, these results were largely influenced by the inhomogeneity of the two groups, particularly due to two outliers that biased the findings. Although this was confirmed in a subgroup analysis that only compared patients with primary instability, definitive conclusions cannot be drawn from this result.

The current study suggests ABR repair is effective/successful to address patients with GLAD lesions, placing the anterior labrum into the defect. As a result, a portion of the glenoid joint surface is sacrificed. To avoid the loss of glenoid joint surface, regenerative cartilage procedures additional to the ABR could be an option. A case series by Lorenz and Scheibel showed good clinical outcomes after treating humeral cartilage defects in the short-term follow-up [24]. However, literature on cartilage regenerative interventions for the glenoid remains scarce.

Nevertheless, consideration should be given to additional concomitant ligamentous injuries besides the Bankart lesion. As an example, humeral avulsions of the glenohumeral ligament have been identified as a risk factor for recurrent glenohumeral instability [25]. Some studies have even advocated for foregoing ABR in cases of HAGL lesions and recommend the Latarjet procedure instead [26,27].

There are several limitations to consider. First, the study design was retrospective, which inherently brings several limitations. Case matching was performed to reduce potential confounding factors as much as possible. Secondly, the present study was statistically underpowered, which increased the risk of a type II error. This was, in particular, due to the follow-up rate of 63.6% (14 out of 22) of the patients with GLAD lesions being available for matching. Third, preoperative Patient-Reported Outcome Measures were not available, preventing any comparisons in this regard. Fourth, a 2-year follow-up may be too short to accurately determine the redislocation rate, as several recent studies have shown that redislocations can occur beyond the first 2 years after surgery.

## 5. Conclusions

Arthroscopic Bankart repair demonstrated favorable mid-term clinical outcomes, low dislocation rates, and high patient satisfaction, irrespective of the presence of a GLAD lesion. Additionally, no difference was detected when differentiating between primary and recurrent instability. However, the follow-up time was heterogeneous, and the follow-up rate was marginal.

## Figures and Tables

**Table 1:** Patient demographics.

Variable	Bankart (*n* = 14)	Bankart + GLAD (*n* = 14)	*p*-Value
Age at surgery (years)	31.9 ± 11.3 (15.3–50.0)	33.9 ± 14.0 (15.0–60.0)	n.s.
BMI	25.0 ± 2.3 (20.3–30.9)	24.2 ± 2.1 (20.3–28.0)	n.s.
Follow-up time (years)	7.9 ± 2.1 (3–11)	5.8 ± 3.1 (2–11)	n.s.
Primary traumatic dislocation (%)	9 (64.3%)	9 (64.3%)	n.s.
Number of anchors	3.2 ± 1.1 (2–4)	2.9 ± 0.5 (1–5)	n.s.

Data are presented as mean ± SD (range) or median (IQR). Statistical significance was defined as *p* < 0.05. BMI, body mass index; GLAD, glenolabral articular disruption; n.s., not significant.

**Table 2:** Patient-Reported Outcome Measures.

Variable	Bankart	Bankart + GLAD	*p*-Value
ASES	97.5 (93.3–100)	100 (96.5–100)	n.s.
ROWE	95.0 (78.8–100)	90 (75.0–100)	n.s.
OSIS	46.0 (43–48)	46.0 (42.0–48.0)	n.s.
WOSI (%)	3.8 (0.8–8.9)	9.0 (3.7– 24.5)	n.s.
Return to sports (weeks)	25.0 (11.3–52.0)	10.5 (7.8–10)	*p* = 0.046
Satisfaction	9.5 (8–10)	9.0 (7.8–10)	n.s.
VAS	0 (0–1.3)	0 (0–1)	n.s.
Level of sport 0–10 preop.	7.5 (6.8–8)	7 (6.9–8.0)	n.s.
Level of sport 0–10 postop.	7.5 (6.8–8.3)	7.0 (4.5–7.6)	n.s.

Data are presented as mean ± SD (range) or median (IQR). Statistical significance was defined as *p* < 0.05; ASES score, American Shoulder and Elbow Surgeons score; BMI, body mass index; GLAD, glenolabral articular disruption; OSIS, Oxford Shoulder Instability Score; n.s., not significant; ROWE, Rowe Score, postop., postoperative; preop., preoperative; VAS, Visual Analogue Scale; WOSI, Western Ontario Shoulder Instability Index.

**Table 3:** Redislocation and Revision Surgeries.

	Bankart	Bankart + GLAD	Total
Redislocation (%)	2 (7.3%)	2 (7.3%)	4 (14.3%)
Revision surgery (%)	2 (3.7%)	0	2 (7.3%)
Open Latarjet procedure (%)	2 (3.7%)	0	2 (3.7%)

Rates of Redislocation and Revision Surgery after ABR in patients with versus without GLAD lesions. ABR, arthroscopic Bankart repair; GLAD, glenolabral articular disruption.

**Table 4:** Demographics and Patient-Reported Outcome Measures of patients with primary instability.

Variable	Bankart (*n* = 9)	Bankart + GLAD (*n* = 9)	*p*-Value
Age at surgery (years)	31.4 ± 12.4 (16.0–51.0)	31.3 ± 11.8 (15.0–49.0)	n.s.
BMI	25.1 ± 2.7 (22.4–30.9)	24.3 ± 2.8 (20.3–28.0)	n.s.
Follow-up time (years)	7.1 ± 2.2 (3–11)	5.9 ± 2.6 (3–9)	n.s.
Number of anchors	3 (2–4)	3 (2–4)	n.s.
ASES	98.3 (84.2–100)	100 (96.7– 100)	n.s.
ROWE	100 (77.5–100)	95 (78.8–100)	n.s.
OSIS	47.0 40–48)	46.5 (44.2–48.0)	n.s.
WOSI (%)	3.3 (0.5–19.8)	8.1 (2.5– 21.3)	n.s.
Return to sports (weeks)	24.0 (10.5–56)	10 (9–16)	*p* = 0.046
Level of sport 0–10, preop.	7.5 (6–8)	7 (7– 8)	n.s.
Level of sport 0–10 postop.	7.5 (7–8)	7.0 (7–8)	n.s.
Satisfaction	10 (8–10)	9.5 (7.5–10)	n.s.
VAS	0 (0–3)	0 (0–1.9)	n.s.
Redislocation (%)	1 (11.1%)	1 (11.1%)	2 (22.2%)

Data are presented as mean ± SD (range) or median (IQR). Statistical significance was defined as *p* < 0.05; ASES score, American Shoulder and Elbow Surgeons score; BMI, body mass index; GLAD, glenolabral articular disruption; ROWE, Rowe Score; n.s., not significant; OSIS, Oxford Shoulder Instability Score; postop., postoperative; preop., preoperative; VAS, Visual Analogue Scale; WOSI, Western Ontario Shoulder Instability Index.

## Data Availability

Due to ethical requirements, the data presented in this study are available from the corresponding author only upon request.

## Abbreviations

ABR	Arthroscopic Bankart Repair
ASES	American Shoulder and Elbow Surgeons Score
BMI	Body Mass Index
FU	Follow-Up
GLAD	Glenolabral Articular Disruption
OSIS	Oxford Shoulder Instability Score
RTS	Return to Sports
VAS	Visual Analogue Scale
WOSI	Western Ontario Shoulder Instability Index

## References

[1] Nazzal E.M., Zsidai B., Pujol O., Kaarre J., Curley A.J., Musahl V. (2022). Considerations of the Posterior Tibial Slope in Anterior Cruciate Ligament Reconstruction: A Scoping Review. Curr. Rev. Musculoskelet Med..

[2] Owens B.D., Dawson L., Burks R., Cameron K.L. (2009). Incidence of shoulder dislocation in the United States military: Demographic considerations from a high-risk population. J. Bone Jt. Surg. Am..

[3] Zacchilli M.A., Owens B.D. (2010). Epidemiology of shoulder dislocations presenting to emergency departments in the United States. J. Bone Jt. Surg. Am..

[4] Eren T.K., Kaptan A.Y., Bircan R., Tosun M.F., Kanatlı U. (2023). Lesion prevalence and patient outcome comparison between primary and recurrent anterior shoulder instability. J. Shoulder Elb. Surg..

[5] Duchman K.R., Hettrich C.M., Glass N.A., Westermann R.W., Wolf B.R., Baumgarten K., Bishop J., Bravman J., Brophy R., Carpenter J. (2018). The Incidence of Glenohumeral Bone and Cartilage Lesions at the Time of Anterior Shoulder Stabilization Surgery: A Comparison of Patients Undergoing Primary and Revision Surgery. Am. J. Sports Med..

[6] Neviaser T.J. (1993). The GLAD lesion: Another cause of anterior shoulder pain. Arthroscopy.

[7] O’Brien J., Grebenyuk J., Leith J., Forster B.B. (2012). Frequency of glenoid chondral lesions on MR arthrography in patients with anterior shoulder instability. Eur. J. Radiol..

[8] Alkhatib N., Abdullah A.S.A., AlNouri M., Ahmad Alzobi O.Z., Alkaramany E., Ishibashi Y. (2022). Short- and long-term outcomes in Bankart repair vs. conservative treatment for first-time anterior shoulder dislocation: A systematic review and meta-analysis of randomized controlled trials. J. Shoulder Elb. Surg..

[9] Bankart A.S.B. (2005). The pathology and treatment of recurrent dislocation of the shoulder-joint. Br. J. Surg..

[10] Murphy A.I., Hurley E.T., Hurley D.J., Pauzenberger L., Mullett H. (2019). Long-term outcomes of the arthroscopic Bankart repair: A systematic review of studies at 10-year follow-up. J. Shoulder Elb. Surg..

[11] Rossi L.A., Pasqualini I., Huespe I., Brandariz R., Fieiras C., Tanoira I., Ranalletta M. (2023). A 2-Year Follow-up May Not be Enough to Accurately Evaluate Recurrences After Arthroscopic Bankart Repair: A Long-term Assessment of 272 Patients With a Mean Follow-up of 10.5 Years. Am. J. Sports Med..

[12] Pogorzelski J., Fritz E.M., Horan M.P., Katthagen J.C., Provencher M.T., Millett P.J. (2018). Failure following arthroscopic Bankart repair for traumatic anteroinferior instability of the shoulder: Is a glenoid labral articular disruption (GLAD) lesion a risk factor for recurrent instability?. J. Shoulder Elb. Surg..

[13] Wermers J., Schliemann B., Raschke M.J., Dyrna F., Heilmann L.F., Michel P.A., Katthagen J.C. (2021). The Glenolabral Articular Disruption Lesion Is a Biomechanical Risk Factor for Recurrent Shoulder Instability. Arthrosc. Sports Med. Rehabil..

[14] Davey M.S., Hurley E.T., Colasanti C.A., Scanlon J.P., Gaafar M., Hogan B.A., Pauzenberger L., Mullett H. (2020). Clinical Outcomes of Patients With Anterior Shoulder Instability and Glenolabral Articular Disruption Lesions: A Retrospective Comparative Study. Am. J. Sports Med..

[15] Elrick B.P., Arner J.W., Horan M.P., Ruzbarsky J.J., Rakowski D.R., Dekker T.J., Goldenberg B.T., Millett P.J. (2022). Concomitant Glenolabral Articular Disruption (GLAD) Lesion is Not Associated With Inferior Clinical Outcomes After Arthroscopic Bankart Repair for Shoulder Instability: A Retrospective Comparative Study. Arthrosc. Sports Med. Rehabil..

[16] Orner C.A., Bastrom T.P., Pennock A.T., Edmonds E.W. (2023). Clinical Outcomes of Adolescents with Anterior Shoulder Instability and Glenolabral Articular Disruption Lesions Compared With Isolated Bankart Lesions. Orthop. J. Sports Med..

[17] Sugaya H., Moriishi J., Dohi M., Kon Y., Tsuchiya A. (2003). Glenoid Rim Morphology in Recurrent Anterior Glenohumeral Instability. JBJS.

[18] Di Giacomo G., Itoi E., Burkhart S.S. (2014). Evolving concept of bipolar bone loss and the Hill-Sachs lesion: From “engaging/non-engaging” lesion to “on-track/off-track” lesion. Arthroscopy.

[19] Richards R.R., An K.-N., Bigliani L.U., Friedman R.J., Gartsman G.M., Gristina A.G., Iannotti J.P., Mow V.C., Sidles J.A., Zuckerman J.D. (1994). A standardized method for the assessment of shoulder function. J. Shoulder Elb. Surg..

[20] Kirkley A., Griffin S., McLintock H., Ng L. (1998). The development and evaluation of a disease-specific quality of life measurement tool for shoulder instability. Am. J. Sports Med..

[21] Rowe C.R., Patel D., Southmayd W.W. (1978). The Bankart procedure: A long-term end-result study. J. Bone Jt. Surg. Am..

[22] Dawson J., Fitzpatrick R., Carr A. (1999). The assessment of shoulder instability. The development and validation of a questionnaire. J. Bone Jt. Surg. Br..

[23] Oenning S., Wermers J., Taenzler S., Michel P.A., Raschke M.J., Christoph Katthagen J. (2024). Glenoid Concavity Affects Anterior Shoulder Stability in an Active-Assisted Biomechanical Model. Orthop. J. Sports Med..

[24] Lorenz C.J., Freislederer F., Salzmann G.M., Scheibel M. (2021). Minced Cartilage Procedure for One-Stage Arthroscopic Repair of Chondral Defects at the Glenohumeral Joint. Arthrosc. Tech..

[25] Longo U.G., Rizzello G., Ciuffreda M., Locher J., Berton A., Salvatore G., Denaro V. (2016). Humeral Avulsion of the Glenohumeral Ligaments: A Systematic Review. Arthroscopy.

[26] Agneskirchner J.D., Lafosse L. (2014). Transfer of the coracoid process in recurrent anterior instability of the shoulder joint. The arthroscopic Latarjet procedure. Oper. Orthop. Traumatol..

[27] Domos P., Gokaraju K., Walch G. (2022). Long-term Outcomes After the Open Latarjet Procedure for the Surgical Management of Humeral Avulsion of the Glenohumeral Ligament Lesions. Am. J. Sports Med..

